# RECOMMENDATIONS TO IMPROVE NURSING HOME QUALITY: A DISCUSSION OF THE 2022 NASEM REPORT

**DOI:** 10.1093/geroni/igac059.226

**Published:** 2022-12-20

**Authors:** Jasmine Travers

## Abstract

Every year approximately four million persons receive care in approximately 15,000 nursing homes across the U.S. The costs of this care exceed $168 billion per year and are projected to grow to $274 billion by 2024. Nursing homes have long been plagued by problems with quality of care.In 1986, the Institute of Medicine released, Improving the Quality of Care in Nursing Homes, a landmark report that fundamentally changed the nation’s approach to nursing home operation and regulation. However, three decades later, significant challenges with nursing home quality remain, many of which were brought to light during the COVID-19 pandemic. As a result, in 2020, the National Academies of Sciences, Engineering, and Medicine (NASEM) convened a panel of 17 experts in nursing home care to examine our nation’s approach to nursing home care, including clinical care, staffing, financing and payment, and regulation, with the goal of making recommendations for improving the quality of care in today’s nursing homes and ensuring the safety and well-being of nursing home residents and staff. Expert members represented areas of diversity, policy, regulation, education, technology, quality measurement and reporting, and clinical practice. The release of this report is April 2022.In this symposium, four committee members will present the main findings and recommendations from the forthcoming and highly-anticipated report, with a particular focus on care delivery, workforce, quality assurance & policy, and equity. During this session, an emphasis will be placed on how the presented recommendations can be incorporated into policy and practice.

